# Furby: fuzzy force-directed bicluster visualization

**DOI:** 10.1186/1471-2105-15-S6-S4

**Published:** 2014-05-16

**Authors:** Marc Streit, Samuel Gratzl, Michael Gillhofer, Andreas Mayr, Andreas Mitterecker, Sepp Hochreiter

**Affiliations:** 1 Institute of Computer Graphics, Johannes Kepler University Linz, Austria; 2 Institute of Bioinformatics, Johannes Kepler University Linz, Austria

**Keywords:** Biclustering, Visualization, Visual Analysis, Soft Clustering

## Abstract

**Background:**

Cluster analysis is widely used to discover patterns in multi-dimensional data. Clustered heatmaps are the standard technique for visualizing one-way and two-way clustering results. In clustered heatmaps, rows and/or columns are reordered, resulting in a representation that shows the clusters as contiguous blocks. However, for biclustering results, where clusters can overlap, it is not possible to reorder the matrix in this way without duplicating rows and/or columns.

**Results:**

We present *Furby*, an interactive visualization technique for analyzing biclustering results. Our contribution is twofold. First, the technique provides an overview of a biclustering result, showing the actual data that forms the individual clusters together with the information which rows and columns they share. Second, for fuzzy clustering results, the proposed technique additionally enables analysts to interactively set the thresholds that transform the fuzzy (soft) clustering into hard clusters that can then be investigated using heatmaps or bar charts. Changes in the membership value thresholds are immediately reflected in the visualization. We demonstrate the value of Furby by loading biclustering results applied to a multi-tissue dataset into the visualization.

**Conclusions:**

The proposed tool allows analysts to assess the overall quality of a biclustering result. Based on this high-level overview, analysts can then interactively explore the individual biclusters in detail. This novel way of handling fuzzy clustering results also supports analysts in finding the optimal thresholds that lead to the best clusters.

## Background

Making sense of large, multi-dimensional data is challenging. Cluster analysis is widely used to discover patterns in such data. In general terms, clustering algorithms group similar objects into clusters such that the clusters themselves are as homogenous as possible and as dissimilar as possible to other clusters. Clustering is often applied to, for instance, gene expression matrices [1,2], consisting of genes (rows) and samples (columns). We need to differentiate between one-way, two-way, and biclustering. In *one-way clustering*, the goal is to determine either clusters in the row or the column dimension. Examples for one-way clustering algorithms are k-means, hierarchical clustering, and affinity propagation [3]. In *two-way clustering*, the result of two sequentially performed one-way clustering runs - one in the row and one in the column dimension - are combined into one result.

### Biclustering

*Biclustering *[4], also known as co-clustering or two-mode clustering, is an emerging field of machine learning. In contrast to one-way and two-way clustering, biclustering is a category of algorithms in which the rows and columns are clustered simultaneously. Biclustering is also different from standard clustering because rows and columns may have multiple or no memberships. In this work, we focus on the visual analysis of biclustering results, as the characteristics of overlapping clusters pose a yet unsolved challenge for visualization.

The array of available biclustering methods ranges from algorithms that try to find a single bicluster, to algorithms that seek to find multiple overlapping biclusters. Madeira and Oliveira [5] surveyed different biclustering algorithms with respect to the structure of their output. Bicluster algorithms are often used to analyze gene expression data [6]. In the context of gene expression, a bicluster may correspond to a pathway that is activated in particular samples (the column members) and that contains certain genes (the row members). Each gene may belong to one bicluster, to more than one bicluster, or to no bicluster at all. The same holds for samples.

In general, clustering algorithms can additionally be differentiated by the kind of memberships they produce. In *hard clustering*, rows and columns are assigned to clusters in a binary way, i.e., they either belong to clusters or not. In *soft clustering*, the result consists of non-binary membership values that describe to what degree rows and columns belong to the clusters. As the assignment of rows and columns to clusters is fuzzy, this is also known as *fuzzy clustering *[7,8].

### Bicluster visualization

**Figure 1:**
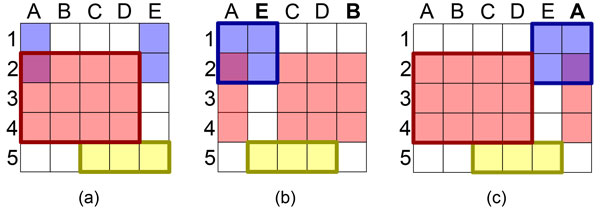
**Biclustering example with three overlapping clusters illustrating the reordering problem**. (a) shows the original matrix where the red and the yellow bicluster form contiguous blocks (thick borders), but the blue bicluster is split into two unconnected blocks. (b) By reordering the columns, the blue bicluster becomes contiguous, however, the red bicluster now gets split up. (c) shows how the duplication of a column solves the ordering problem.

Let us consider the visualization of hard clustering results first. In order to understand and interpret hard clustering results, it is necessary to visualize the clusters together with the underlying data. Clustered heatmaps are the standard technique for visualizing both one-way and two-way clustering results. In clustered heatmaps, the rows or columns are reordered, such that clusters can be recognized as contiguous blocks consisting of adjacent cells. Showing clusters as contiguous blocks is highly desired, as it simplifies the detection and interpretation of patterns. However, for biclustering results, where clusters can overlap, rearranging the matrix this way is often impossible. Let us consider the example from Figure 1 that shows a 5x5 matrix with three clusters. In Figure 1(a), the columns are sorted such that the red and yellow clusters are represented as contiguous blocks, as indicated by a thick border. However, this sorting splits the blue cluster into two unconnected blocks. In Figure 1(b), columns B and E are swapped, which makes it possible to show the blue cluster as a contiguous block, but splits the red cluster. Consequently, even in small matrices there is often no optimal order of rows and columns where all clusters form contiguous blocks. The sorting problem can be solved by duplicating rows and/or columns, as demonstrated in Figure 1(c). However, the duplication approach does not scale, as it potentially produces large output matrices for comparably small input matrices.

Interpreting biclustering results is often time-consuming and tedious, as it is usually done statically by visually inspecting many separate plots. Adding fuzzy clustering to this equation makes the situation even more difficult.

Fuzzy biclustering is a visualization research problem that cannot be addressed by any of the existing tools. We will first elaborate on how biclustering results can be represented and then introduce the FABIA fuzzy biclustering algorithm [9]. We use FABIA to demonstrate the proposed technique; however, note that any other biclustering algorithm that produces overlapping clusters can be used in the same way. We continue by introducing general requirements for bicluster visualization, which we use to review existing work in this field. We then present *Furby*, an interactive visualization technique for analyzing fuzzy biclustering results. After a brief description of the implementation, we present how the tool can be used effectively to analyze a real-world dataset. Before concluding the paper, we discuss the scalability of our tool to large datasets.

### Representation of biclustering results

Biclustering data can generally be represented by three matrices: *X, L*, and *Z*. The *X *matrix represents the input data to be clustered. The biclustering results are represented by *L *and *Z*. The *L *matrix contains the relationship information between rows and biclusters, and the *Z *matrix contains the same information for columns. While for hard biclustering results, *L *and *Z *hold binary values (1 = row/column is part of a cluster, 0 = row/column is not part of a cluster), for fuzzy biclustering results they contain real values that denote the degree of membership, starting with 0, which means that the row or column does not belong to the considered bicluster.

### FABIA biclustering algorithm

*FABIA *[9] is an established biclustering algorithm which has been successfully applied not only to drug discovery and systems biology, but also to enhance recommender systems. FABIA, as a generative model, is based on factor analysis, but can be considered as a sparse matrix decomposition algorithm. As described above, the observed data matrix *X *is decomposed into two matrices: the *L *matrix describes memberships of rows (genes) to biclusters, and the *Z *matrix describes the memberships of columns (samples) to biclusters. Consequently, a bicluster is described by row and column memberships. The FABIA model assumes that biclusters have only few row and column members. This is the typical situation for gene expression data, where pathways contain only few genes (compared to all genes), which are activated in only few samples. This situation is also typical for recommender systems, where a customer buys only few products, and a certain product combination is chosen only by few customers. Another example is word-document matrices, where a bicluster is a certain topic (a document contains few topics and a topic contains few indicative words). Thus, in all these applications the data matrix is sparse, as are the matrix that describes row memberships, and the matrix that describes column memberships. In FABIA models, this sparsity is reflected by sparse row and column decomposition matrices enforced by sparse priors in a Bayesian framework. FABIA describes row and column memberships by real numbers. Hence, the bicluster memberships are fuzzy, and it is difficult to decide in the "twilight zone" whether a column or a row indeed belongs to a bicluster.

The memberships must often be inspected visually by an expert, who then decides how good the bicluster pattern is (gene pattern of a pathway) and how strong the signal is (gene expression). Assessing the relationship between biclusters is an even more complex task. Do two biclusters partially represent the same information? If yes, which columns and/or rows do they share? To comprehend the information in the biclusters and their mutual dependencies, a visual representation of this information is highly desired.

### Requirements

Based on interviews with domain experts and surveying the body of previous work, we have elicited seven requirements that an effective fuzzy bicluster visualization needs to fulfill. We will assess existing work in this field against these requirements. Later sections will demonstrate how our technique addresses these requirements.

• R I: Show individual biclusters

As the primary goal of data clustering is to find data subsets that are similar in some respect, the most basic requirement for a bicluster visualization technique is to present the individual biclusters to the analyst. The visualization needs to encode the data elements that form the cluster, together with the corresponding column and row identifiers. An effective visualization of a single cluster is essential for interpreting the data.

• R II: Visualize shared rows and columns of multiple biclusters

In a biclustering result, columns and rows can be assigned to multiple biclusters. For interpreting the clustering result, it is important to communicate which rows and columns are shared between which clusters. This is also relevant to identifying similar clusters, i.e., a set of clusters with a large overlap.

• R III: Visualize membership of rows and columns to biclusters

In contrast to requirement **R I**, where users are interested in a single bicluster in detail, analysts also want to investigate to which biclusters a single row or column is assigned to.

• R IV: Scalability

A well-designed bicluster visualization should scale to large datasets, to many biclusters, and to a large number of shared rows and columns between biclusters.

• R V: Visualize bicluster strength

When visualizing fuzzy biclustering results, it is important to encode the membership values of rows and columns to biclusters. The membership value represents to what degree a row or column belongs to a particular cluster. By setting thresholds, fuzzy clusters can be transformed into hard clusters. Encoding the membership value of rows and columns in addition to the raw data, allows analysts to judge the strength of clusters.

• R VI: Interactive cluster refinement

Supporting analysts in the process of transforming fuzzy clusters into hard clusters by setting thresholds for the membership values is a central task of fuzzy bicluster visualization. Analysts need to be able to set the threshold interactively and immediately see the resulting hard biclusters. The combination of interactive refinement and encoding of shared rows and columns should help the analyst to determine optimal membership threshold values.

• R VII: Visualize relationships to additional metadata

An effective bicluster visualization should allow analysts to relate rows and columns of biclusters to additional external data. For example, analysts want to investigate the correlation of biclusters defined on gene expression data with, for instance, patient groups, cancer subtypes, or tumor staging.

### Related work on cluster visualization

The key to let analysts gain new insights in large and complex multi-dimensional datasets is to combine the strength of automated algorithmic techniques with the power of interactive visualization [10-13]. Numerous techniques for the interactive visual analysis of clustering results have been proposed over the last years.

In order to discuss interactive cluster visualization techniques, we split up the body of existing work according to types of clustering. The standard technique for oneand two-way clustering is the clustered heatmap, where rows and/or columns are reordered to reflect the similarities. Examples for visual analysis tools that provide interactive heatmaps are Mayday [14], Caleydo [15,16] and the Dual Analysis framework [17]. For hierarchical clustering results, the clustered heatmap is commonly extended with a dendrogram that represents the similarities between the rows or columns [18]. The Hierarchical Cluster Explorer (HCE) [19] and MultiClusterTree [20] are both approaches that allow interactive analysis of hierarchical clustering results.

However, as mentioned at the beginning of the Bicluster visualization section, for biclustering results it is often not possible to rearrange the matrix in order to represent all clusters as contiguous blocks (see Figure 1), which is essential for interpreting the clusters. A simple approach to visualizing biclustering results is to create a separate plot for each bicluster, as implemented, for instance, in the Biclustering Analysis Toolbox (BicAT) [21], the BiClust R toolbox [21] and the BiVisu tool [22]. Showing every cluster as a separate plot allows analysts to inspect the clusters individually, which addresses requirement **R I**. However, this makes it impossible to see which rows and columns they share, which violates **R II**. Jin et al. [23] formulated the reordering issue as an optimization problem and proposed a reordering approach by exploiting analogies to the hypergraph vertex ordering problem. Grothaus et al. [24] propose to duplicate rows and columns to resolve situations where reordering is not possible. The BiCluster viewer [25] follows the same approach, but additionally allows analysts to interactively decide which clusters to show contiguously in order to minimize the number of duplicates. As this can, however, still result in very large matrices, scalability is limited (see **R IV**).

The work that is probably related most closely to ours is the BicOverlapper tool [26], which presents the biclustering result in a multiple-coordinated view setup. A parallel coordinates view and a heatmap show the individual biclusters, realizing **R I**. The overlapper view visualizes the bicluster network as a force-directed graph where biclusters are represented as overlapping groups. Although the BicOverlapper tool encodes the overlaps between clusters (**R II**) and the cluster assignment (**R III**), it does not scale well to many biclusters (**R IV**), as it creates occlusion problems, which renders obtaining an overview of the biclustering results as a whole impossible.

Only a small number of articles on visualizing fuzzy clustering results have been published. Most of them propose extensions to classical clustering visualizations, including parallel coordinate plots [27], heatmaps [28], and RadVis [29] - a radial visualization technique, in which membership values are projected to polar coordinates. A similar approach was developed by Rueda and Zhang [30], which maps membership values to a hyper-tetrahedron in the 2D or 3D space representing three or four fuzzy clusters. clusterMaker [31] takes a different approach by representing a one-way fuzzy clustering result as a force-directed graph where the clustered entities are shown as nodes and color is used to encode the cluster membership. However, all these methods focus on the membership or membership values of the rows and columns and ignore the underlying data of the clustering result (see **R I**).

In summary, none of the existing approaches are able to address the requirements in a satisfactory way. In particular, the visualization of fuzzy biclustering seems to be a blank area in the research map - a blank which Furby attempts to fill.

## Methods

Before we introduce the Furby visualization technique, we first discuss the typical workflow an analyst follows when analyzing a biclustering result:

• 1 Gain overview of clusters

The analyst starts by inspecting the overall cluster network. Measures for assessing the relevance of the biclusters are the patterns of the individual clusters and the number of rows and columns they share. The most interesting clustering results may, for instance, be characterized by a high homogeneity of the elements within the clusters and by a small overlap between clusters. However, depending on the application domain and the task at hand, also large overlaps could be of interest to the analyst.

• 2 Globally adjust the threshold of the bicluster membership values

In the case of fuzzy clustering, the analyst globally adjusts the threshold that transforms the fuzzy clusters into hard clusters. The result of the threshold tuning should be reflected immediately in the visualization. This step is optional.

• 3 Inspect individual clusters in detail

The analyst then explores clusters that she has identified as potentially interesting in the overview visualization. In order to interpret the meaning and biological relevance of a single cluster, the analyst examines its elements in detail - including additional metadata.

• 4 Locally adjust the thresholds of bicluster membership values

In contrast to a global threshold adjustment, the analyst refines the local thresholds for single biclusters (without changing the global thresholds that are applied to all other clusters). This step is optional.

**Figure 2:**
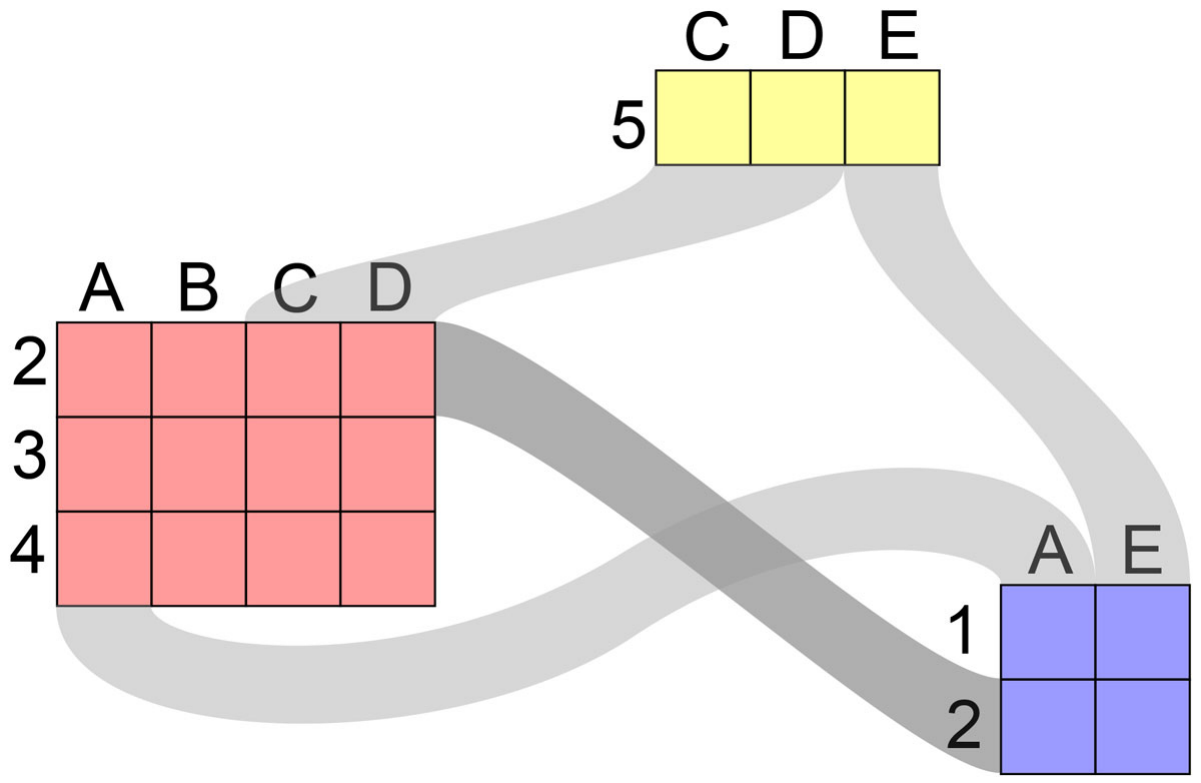
**Bicluster visualization concept showing the same sample matrix as in Figure 1**. The clustering result is shown as a graph, in which nodes correspond to biclusters and edges between the nodes encode rows and columns shared by the clusters.

To realize this workflow, Furby follows an *overview+detail *approach. In this section, we first introduce the *cluster network overview*, where each bicluster is represented as one node in a graph. Figure 2 illustrates this concept using the same sample matrix as in Figure 1. The edges in the graph represent the rows and columns overlapping between the clusters. In the second part of this section, we focus on the *detail view*, which enables analysts to explore single biclusters and their elements.

### Cluster network overview

**Figure 3:**
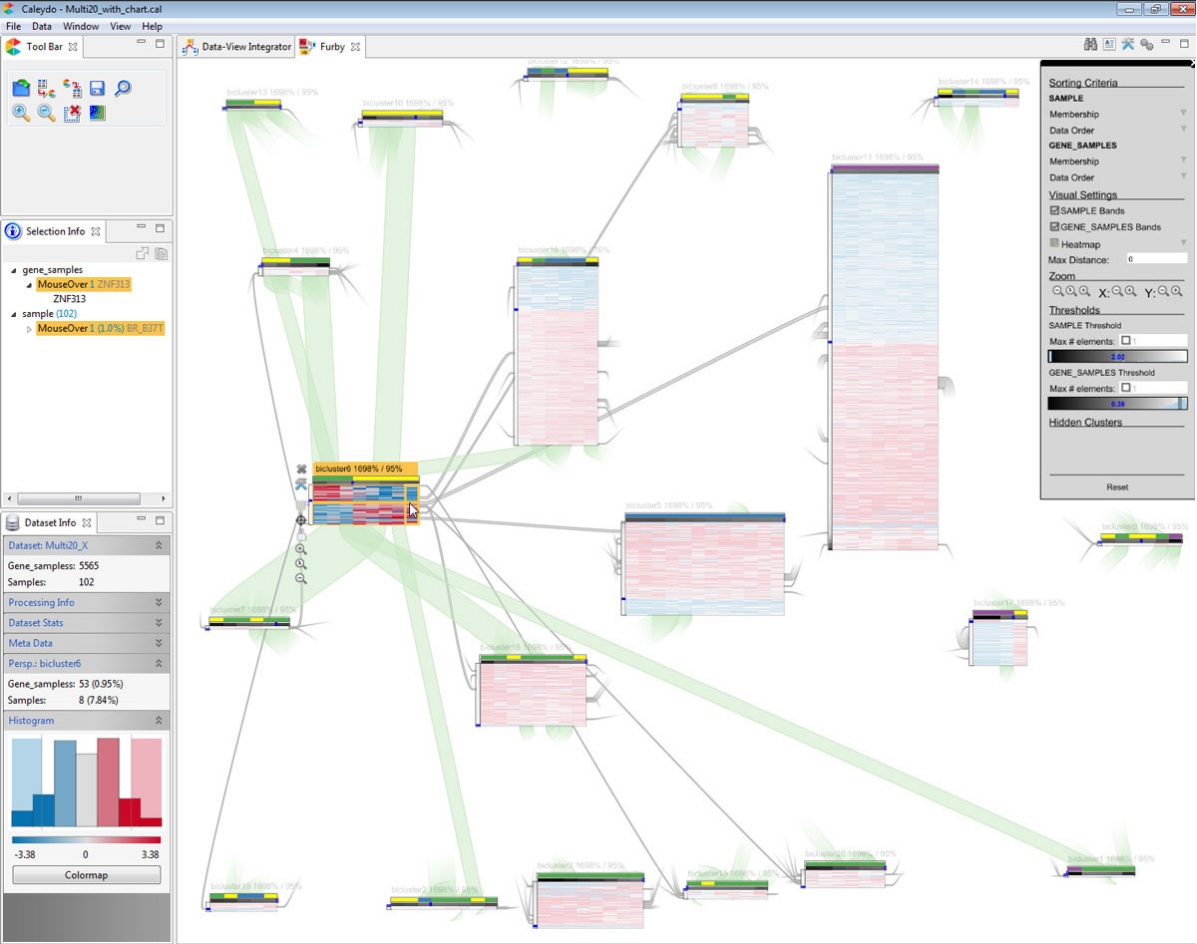
**Overview visualization of a biclustering result with 20 clusters**. Grey bands show the overlap in gene dimension and green bands visualize the relationsships in sample dimension. The analyst selected *bicluster6 *to focus on the overlap between the selected and all other clusters.

The overview visualization presents the biclustering result as a graph in which individual clusters are the nodes and the rows and columns overlapping between the clusters are the edges. Figure 3 shows an example biclustering result with 20 clusters. We layout the graph using a force-directed algorithm [32] in which overlapping clusters attract each other. The more rows and columns two bicluster share, the bigger is the attracting force. By default, all biclusters repulse each other, resulting in a layout in which clusters with a large overlap form groups.

Bicluster nodes in the graph represent the data as a heatmap, addressing requirement **R I**. By default, we apply a red-grey-blue color scheme. However, analysts can change and refine the color mapping on the fly during the analysis. The overlaps between biclusters are encoded using bands connecting the biclusters, which satisfies requirement **R II**. In previous work [33,34], we have already made use of bands to visualize the relationships between clusters represented as heatmaps in the context of one-way clustering. In Furby, the same approach is applied in both dimensions, rows and columns. The thickness of the bands is proportional to the number of rows and columns shared by the clusters. The bands are attached to the bicluster heatmaps at the position of the shared rows and columns within the heatmap.

#### Selection and highlighting

Furby supports *linking & brushing*. Hence, when the user selects one or more rows and columns, all corresponding instances within all clusters and bands are automatically highlighted. This helps analysts to identify how often individual rows and columns are contained in the clusters (see requirement **R III**).

**Figure 4:**
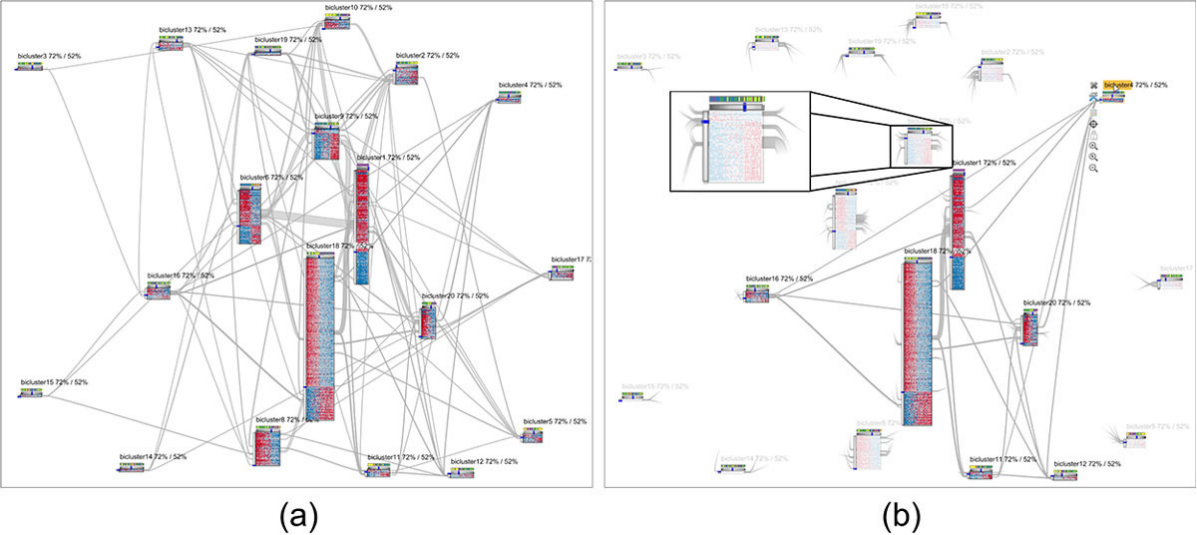
**Bicluster neighborhood visualization**. (a) shows the regular cluster network visualization that enables users to gain an overview of the full bicluster network. In (b), the user has selected a bicluster, causing all unconnected biclusters to be faded out. Edges connecting faded out clusters are replaced by stubs pointing in the direction of the bicluster they connect.

Keeping the visual clutter to a minimum and letting the analyst focus on the currently selected cluster are important aspects of a scalable visualization technique, formulated in requirement **R IV**. To achieve this, we use a combination of stubs [35,36] and fading effects. Stubs are small indicators that replace the bands and point in the direction of the connected cluster, resulting in a significant reduction of visual clutter. While Figure 4(a) shows a screenshot of the regular overview, Figure 4(b) shows the adapted version in which the analyst hovers over a single cluster heatmap. All unconnected clusters within a distance of N hops in the underlying graph are faded out, and in a similar fashion all unconnected bands are replaced by stubs. Using stubs instead of bands lets analysts focus on the local graph neighborhood of the selected cluster of interest. Note that the distance variable N can be manipulated interactively, which allows the user to take smaller or larger portions of the cluster network into account.

In addition to direct interaction with the biclusters, analysts can globally manipulate parameters using a toolbar shown on the right side of the interface (see Figure 3). The toolbar allows users to turn off bands in both dimensions (rows and columns), for instance.

#### Navigation and zoom interaction

We automatically calculate the initial zoom settings of the overview visualization, such that the aspect ratio of the bicluster heatmaps is on average 1 and that the total space occupied by the clusters does not exceed a certain maximum. This ensures that the visualization of biclustering results obtained from diverse datasets produces acceptable results for various screen resolutions. In addition to this initial adjustment of the scaling, analysts can adapt the zoom factor of a single as well as of all biclusters via zoom controls in the local cluster specific toolbar and the global toolbar, respectively. The zoom factors can be manipulated for both dimensions independently. This is useful, since biclusters might have a distorted aspect ratio, depending on the raw data and the quality of the result. In addition to the zoom controls in the toolbars, mouse shortcuts can be used. Holding the CTRL key while applying the mouse wheel zooms both dimensions simultaneously, holding the SHIFT key just the vertical dimension, and holding just the ALT key the horizontal dimension. If the analysts moves the mouse over a heatmap during the zoom interaction, the scaling will only be applied to this particular cluster - otherwise all clusters will be scaled simultaneously.

### Detail visualization

**Figure 5:**
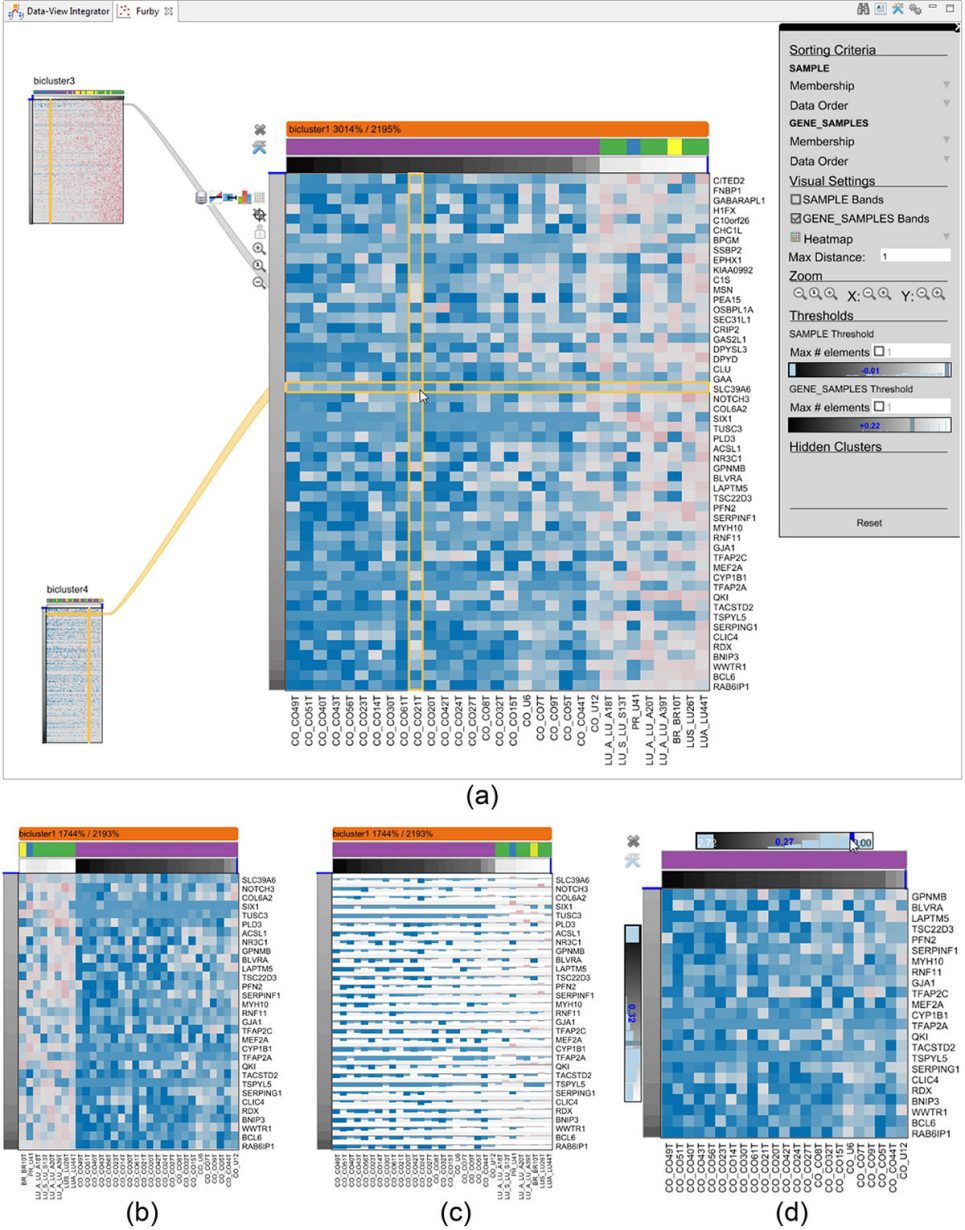
**Visualization of single biclusters**. (a) shows the bicluster as a heatmap where the rows and columns are sorted by membership value. In (b), the columns are sorted by an additional categorical variable. (c) shows the same data as in (a) and (b) represented as a bar chart. In (d), the analyst has adjusted the membership value thresholds.

In contrast to the overview visualization described in the previous section, the detail view focuses on a single bicluster and allows an in-depth exploration of its elements, addressing requirement **R I**. Analysts can bring a bicluster into the focus by doubleclicking its header, which shows its name. The bicluster will then be scaled up and put in the center of the visualization. The directly connected neighbor clusters are shown as thumbnails in the remaining area. The neighborhood degree, whether a bicluster should be shown in addition to the focused bicluster, will be defined again by the maximum distance parameter N, which can be specified in the toolbar (see Section Selection and highlighting). Figure 5(a) shows an example where N is set to 1. By setting N to 0, only the detail bicluster remains visible, which is useful when the context of a cluster is not of current interest to the user. In the detail mode, analysts can browse through the biclusters by using the left and right arrow keys. By double-clicking the header again, the visualization switches back to the overview cluster network.

The main area of a bicluster node is a multiform visualization, i.e., the applied visualization technique can be switched on demand. By default, we present the bicluster data as a heatmap, as it is the most commonly used technique for this kind of data. Besides the heatmap representation, we provide additional visualization techniques, including a bar chart and a histogram for all values. To ensure the readability of the identifier labels, we use a combination of scrollbars as well as orthogonal stretching, to magnify selected rows and columns. In addition to the actual data shown in the cluster visualization, we add extra rows and columns to visualize metadata as well as membership value information in case of fuzzy clustering results, as described in the next two sections.

### Interactive membership value adjustment

Fuzzy biclustering produces soft memberships instead of binary cluster assignments, determining to what degree rows and columns belong to a bicluster. However, to visualize a bicluster, a certain membership value threshold needs to be chosen - this is the process of transforming a fuzzy clustering result into hard clusters. Furby allows analysts to interactively manipulate these thresholds, addressing requirement **R VI**. Thresholds can be defined independently per dimension, locally per bicluster, or globally via the toolbar. Analysts can use a slider to manipulate the thresholds, while the background of the sliders show a histogram of the underlying membership values. In addition to specifying a threshold value, users can restrict the bicluster to only contain the top M rows or columns.

We show the membership value for each row and column as an additional greyscale bar that is directly attached to the bicluster visualization, as shown in Figure 5. The darker the value, the higher the membership value. This membership value indicator addresses requirement **R V**. Some bicluster algorithms, including FABIA, produce positive as as well as negative membership values. We indicate 0 by a blue line.

### Adding metadata

Often, users want to analyze the clusters in the context of externally loaded metadata, as described in requirement **R VII**. We enable analysts to load categorical or ordered numerical metadata for rows and columns. Examples for additional data defined on biological samples are gender, age, and tumor staging. In gene dimension, analysts can load metadata such as Gene Ontology (GO) terms [37] and the results of a gene set enrichment analysis [38] performed on KEGG pathways [39]. Furthermore, external cluster assignments can be loaded as additional categories to compare multiple clustering results.

In Furby, we visualize contextual data by attaching additional bars to the cluster visualization. In the case of categorical data, we assign a unique color to each category. For ordered numerical data, analysts can choose from a set of pre-defined color schemes. When the user hovers over an additional metadata bar, we show the name of the category as a tooltip.

### Sorting strategy

We enable analysts to define a primary and a secondary sorting criterion for both the rows and columns. The secondary sorting criterion is only used if the first one produces a tie. The sorting is applied to both the overview and the detail visualization. Furby supports the following sorting criteria:

**• data order**: takes the original order from the raw data files

**• additional metadata**: sort by an external metadata annotation

**• membership value**: sort by soft membership values (only for fuzzy biclustering results)

The examples in Figure 5 contain membership value bars in both dimensions and an additional external clustering assignment for the samples. In Figure 5(a) and 5(c), the sample ordering is determined by decreasing membership values, as indicated by the bars from dark to bright. However, this leads to fragmented clustering assignment bars. In Figure 5(b), the sorting is reversed, by grouping the samples according to their external cluster assignment and by the membership value within an assignment category.

## Implementation

The Furby visualization technique is part of *Caleydo*, an open-source data visualization framework [15,16]. Caleydo is implemented in Java and uses OpenGL/JOGL for rendering. A demo version of Furby for Windows, Linux, and Mac OS × is freely available at http://furby.caleydo.org.

### Data loading

We integrated a data importer to simplify the loading of a biclustering result. The data can be loaded from CSV files. Three matrices, *X, L*, and *Z*, are needed to specify a clustering result. The *X *matrix contains the actual data, and the *L *and *Z *matrices hold the membership values. While in hard biclustering the *L *and *Z *matrices contain binary membership values, in fuzzy clustering the values are real numbers, with 0 indicating that rows or columns do not belong to the considered bicluster. Categorial cluster annotations and initial membership threshold guesses can be loaded in addition. Further, we provide an R script [40] for exporting FABIA result objects in the CSV file format required by Caleydo. The script can easily be adapted to load results of arbitrary bicluster algorithms from R.

### Force-directed layout

To simplify and speed up the physical simulation of the forces, we internally use ellipses as shapes instead of the actual rectangular bounding boxes of the bicluster nodes. Nodes are positioned automatically following the force-directed layout approach, but they can also be freely repositioned using drag and drop. This way layout issues can be resolved, especially since we apply a damping factor within the layout algorithm to ensure that the layout quickly stabilizes. While this prevents the drifting of nodes caused by rounding errors of the physical simulation, it can produce sub-optimal layout results. However, according to user feedback, a stable layout is preferred over an optimal one which takes longer to be created. Layout stability is particularly important for users to maintain their mental map.

## Results

We demonstrate the application and usefulness of Furby by a representative analysis of the *multiple tissue types *dataset [41] as provided by Hoshida et al. [42] using a soft biclustering result created by the FABIA algorithm. The dataset contains the gene expression values of 5,565 genes for 102 samples extracted from various types of tissue. In addition, we use the tissue type categorization as metadata annotation, to support the interpretation of the biclusters.

**Figure 6:**
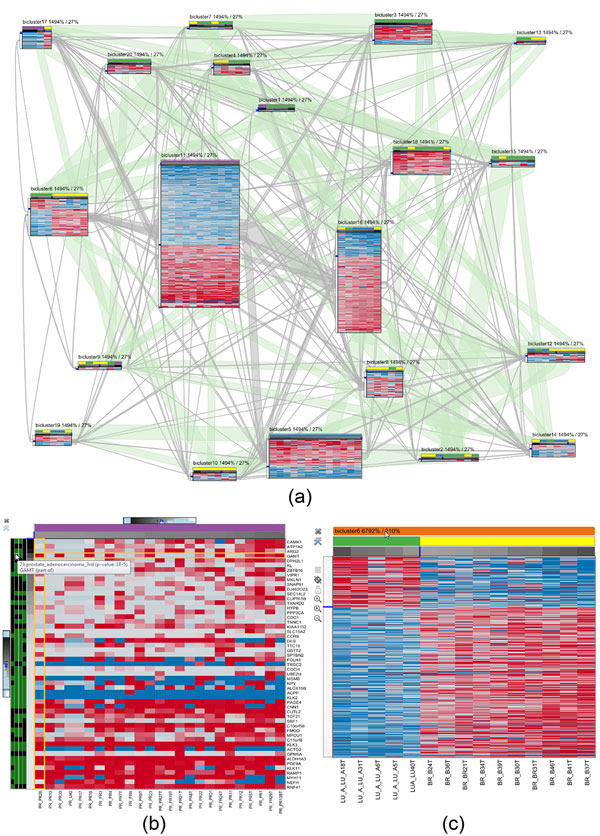
**Intermediate analysis steps in Furby**. (a) The analyst starts by inspecting the overview visualization showing the biclustering result of the *multiple tissue types dataset*. The initially set membership value thresholds result in a large number of overlaps in both dimensions. Consequently, also the visual representation in Furby is very cluttered. By optimizing the global membership thresholds, the analyst gets a much cleaner representation, as shown in Figure 3. In (b), the analyst inspects a bicluster with high membership values in gene and sample dimension. The additional metadata bar in sample dimension encodes the tissue type (purple = prostate). The metadata bar in gene dimension represents the 4 top results of a DAVID functional annotation analysis (green = part of gene set, black = not part of gene set). The cluster in (c) contains lung (green) and breast tissue samples (yellow) that show an inverse gene regulation.

The analyst starts by computing a fuzzy biclustering using the FABIA R package. She then uses the provided R-script for exporting the *X, L*, and *Z *matrices containing the gene expression data and the biclustering result. In addition, the initial membership threshold guesses as well as the tissue type annotations are exported. After loading the data, Furby initially shows the visualization in Figure 6(a). A characteristic of FABIA is that it produces both positive and negative membership values. In Furby, we handle this by using the absolute value for assigning genes and samples to biclusters. This leads to biclusters with four quadrants that are defined by the combination of positive and negative membership values in both dimensions. However, analysts can customize this behavior via the context menu of the threshold slider.

Following the analysis workflow introduced at the beginning of the Methods section, the analyst first tries to gain an overview of the overall biclustering result by inspecting the cluster network. As shown in Figure 6(a), all biclusters have small gene overlaps, except *bicluster16 *and *bicluster11 *which share more genes, as indicated by the thick bands connecting these bicluster nodes. After turning on sample bands, by clicking the corresponding entry in the toolbar, the analyst sees that a large number of samples is connected across the biclusters. However, given the fact that the 20 biclusters are defined on only 102 samples, this is to be expected.

As a next step, she reduces the number of gene overlaps by adjusting the membership value threshold. By increasing the threshold via dragging the global threshold slider to the left, the analyst can observe how the bicluster nodes gradually shrink and the bands become thinner or disappear. Using the zoom feature, the analyst adapts the aspect ratio of the bicluster to avoid distorted bicluster nodes, which yields the visualization shown in Figure 3. Closer examination of the individual clusters reveals that *bicluster11 *has high membership values in sample and gene dimension according to the membership bars, compared to the remaining biclusters. In addition, it is the only bicluster that does not share any samples with other biclusters. *bicluster6 *also attracts the analyst's attention, as it only contains lung and breast tissue samples, while most other biclusters contain a mix of many tissue types. *bicluster11 *and *bicluster6 *are therefore interesting candidates for a detailed inspection.

By double-clicking the header of *bicluster11*, the cluster is enlarged and moved to the center of the visualization. Since related biclusters are not of current interest, the analyst reduces the maximum distance parameter N to 0, result in the visualization depicted in Figure 6(b). Looking at the sample membership value bar, she realizes that the values are homogeneously high, indicating a strong cluster. The analyst then adapts the local threshold of the sample dimension in order to include more samples. However, as this does not include any new samples, the analyst concludes that the cluster is stable in sample dimension and therefore well defined. Moreover, she observes that the samples are exclusively prostate tissue samples. Looking at the gene dimension, she can see that the genes have comparably high membership values. In order to reduce the number of genes in the cluster, she increases the membership level threshold in gene dimension. By inspecting the result of a gene annotation enrichment analysis [43], which is provided as an additonal metadata bar, she infers that the strongest bicluster genes seem to stem from prostate tissue.

The second interesting bicluster, *bicluster6*, only consists of lung (yellow) and breast tissue samples (green), as shown in Figure 6(c). The analyst recognizes that changing the sample order, such that the dimension is sorted by the tissue type first and then by the membership value within the tissue types, would not change the sorting of samples in the bicluster, as all lung tissue samples have a membership value below 0 and all breast tissue samples a value greater than 0. By inspecting the gene expression values, the analyst realizes that the lung tissue samples have an inverted expression compared to the breast tissue samples. The analyst finally concludes that the two tissue types may be regulated by the same pathway.

## Discussion

Analyzing clustering results is a challenging task which our method simplifies by providing a combined solution that shows both overall structure and the details. However, with an increasing number of overlapping biclusters, the layout and bands become increasingly complex. Especially in cases where biclusters share rows and columns at the same time, our approach can result in a suboptimal layout. We address this problem by using stubs to tidy up the visualization and by fading out clusters of the visualization that are not of current interest.

Interactive manipulation of the membership thresholds is a key feature of Furby, as it allows analysts to explore fuzzy biclustering results in an intuitive way. However, changing the thresholds causes clusters to grow or shrink, which in turn also increases or decreases the white space in the layout. Biclusters that are degenerated in size, i.e., which have many more rows than columns or vice versa, are particularly problematic in this respect. This is, however, a regular case when analyzing gene expression data, where the number of genes is typically much higher than the number of samples. These degenerated clusters make it increasingly hard to find a good layout and to determine the routes for the bands between them. We address this issue effectively by calculating a proper initial layout that is optimized for the initial thresholds and by letting analysts interactively adapt the horizontal and vertical zoom factor. Although this solution is not fully automated and requires action from the analyst, it works very well in practice. If the visualization gets excessively crowded and if clusters start to grow into the drawing region of other clusters, the analyst can easily fix the problem by decreasing the zoom factor. On the other hand, if clusters become smaller because rows and/or columns are excluded, the user can fill up the empty space in the layout by increasing the zoom factor.

## Conclusion

In this paper we have presented Furby - an interactive visualization tool for exploring and analyzing fuzzy biclustering results. The incorporation of multiple levels of detail enables analysts to gain an overview of the overall network of clusters and to investigate individual biclusters in detail.

As part of future work, we intend to provide the analyst with basic statistics about clusters, such as variance and skewness, and their overlap, for instance, the Jaccard index as a measure for the similarity of two clusters. In addition to just showing these statistics, it would then be possible to use this information to guide the analyst to potentially interesting aspects in the data. Furthermore, we plan to conduct a user study in order to formally evaluate the effectiveness of the presented approach. We also believe that the proposed visualization technique could be applied in the context of subspace clustering [44,45]. Another interesting avenue for future research is the comparison of multiple biclustering results.

## Competing interests

The authors declare that they have no competing interests.

## Authors' contributions

Marc Streit conceived the project and designed the technique. Marc Streit and Samuel Gratzl wrote the manuscript with contributions from Andreas Mayr, Andreas Mitterecker, and Sepp Hochreiter. Michael Gillhofer and Samuel Gratzl developed the research prototype. Andreas Mayr, Andreas Mitterecker, and Sepp Hochreiter provided continuous feedback during the development.
